# Using PROMs during routine medical consultations: The perspectives of people with Parkinson’s disease and their health professionals

**DOI:** 10.1111/hex.12899

**Published:** 2019-06-14

**Authors:** Olga C. Damman, Marjolein E. A. Verbiest, Suzanne I. Vonk, Henk W. Berendse, Bastiaan R. Bloem, Martine C. de Bruijne, Marjan J. Faber

**Affiliations:** ^1^ Department of Public and Occupational Health, Amsterdam Public Health Research Institute, Amsterdam UMC Vrije Universiteit Amsterdam Amsterdam The Netherlands; ^2^ Radboud Institute for Health Sciences, Scientific Center for Quality of Healthcare (IQ Healthcare) Radboud University Medical Center Nijmegen The Netherlands; ^3^ Tilburg School of Social and Behavioral Sciences, Tranzo Scientific Centre for Care and Welfare Tilburg University Tilburg The Netherlands; ^4^ Department of Neurology, Amsterdam Neuroscience, Amsterdam UMC Vrije Universiteit Amsterdam Amsterdam The Netherlands; ^5^ Department of Neurology, Donders Institute for Brain, Cognition and Behaviour Radboud University Medical Centre Nijmegen The Netherlands

**Keywords:** patient perspective, patient‐reported outcome measures, professional perspective, shared decision making

## Abstract

**Background:**

The use of patient‐reported outcomes measures (PROMs), such as quality of life or symptoms like pain or fatigue, is increasingly embraced within patient‐centred care and shared decision making.

**Objectives:**

To investigate: (a) how patients and health professionals think about using PROMs during routine medical consultations; (b) for which purpose(s), patients and health professionals want to use PROMs during those consultations; and (c) how patients interpret PROMs information presented in various formats. People with Parkinson's disease and their health professionals served as case example.

**Methods:**

We performed semi‐structured interviews with patients (N = 13) and professionals (N = 7 neurologists; N = 7 physiotherapists). We also used a survey in which patients (N = 115) were shown six figures displaying different information types. Presentation formats of this information varied (line/bar graphs). Interpretation by patients, perceived usefulness of information, attitude towards using information during routine medical consultations and (hypothetical) decisions were assessed.

**Findings:**

Patients and professionals were generally positive about using PROMs during medical consultations. Professionals stressed the opportunity to monitor changes in individual PROMs over time. Patients were primarily positive about aggregated PROMs to make treatment decisions. This information was also most often interpreted correctly, especially when presented through a line graph (90.1% correct). Professionals thought patients should take the initiative in discussing PROMs, whereas patients thought professionals should do so.

**Conclusion/Discussion:**

When used in routine medical consultations, PROMs seem to have potential to support shared decision making and facilitate patient‐professional communication. However, training seems needed for both patients and professionals to facilitate actual discussion and proper interpretation.

## 1 INTRODUCTION

Where patient‐reported outcome measures (PROMs) have traditionally been used to evaluate the effectiveness of medical interventions from the perspective of patients,^1^ they are nowadays thought to have a more comprehensive role in health‐care quality improvement.^2^, ^3^, ^4^ PROMs are standardized questionnaires, completed by patients, measuring how they experience their health (ie symptoms, functional status). Well‐known PROMs are quality of life questionnaires, such as the SF‐36 and EQ‐5D. Integrating PROMs within routine clinical practice might increase the motivation of patients to complete PROMs (which currently proves challenging), and such use has also been theorized to improve management and monitoring of patients’ problems.^5^, ^6^ Apart from that, using PROMs in routine medical consultations could improve doctor‐patient communication^7^ and informed patient decisions^4^, ^8^ and as such improve quality of care. At an aggregated level, they can also be used to evaluate and compare treatment options and provider performance.^3^, ^4^, ^9^

Using PROMs during routine medical consultations by health professionals and patients would suit current ideas on person‐centred medicine and shared decision making.^4^, ^10^^.^ Although shared decision making has become the standard in health care to enable patients in making informed decisions,^11^, ^12^, ^13^ the use of PROMs in this context has remained understudied. For patients who must make choices between different treatment options and health‐care providers, it is, however, not only important to have insight into clinical outcomes (eg survival rates), but also into how different treatment options may impact on their own health and quality of life, as viewed by patients themselves. Previously, patients have been positive about using information derived from PROMs, especially because it may prompt discussions about actual health issues with professionals.^14^ However, patients do not find all PROMs equally relevant or even bothersome or unnecessarily confronting, and such perceptions may well hinder the adoption of PROMs into daily clinical practice. Several previous studies have therefore involved patients into developing PROMs.^10^, ^15^ Also, studies have shown that meaningful and comprehensible presentation of PROMs data is important to help patients with interpreting the data correctly and with using the data in important medical decisions, such as choosing a particular type of treatment or health‐care provider.^16^, ^17^, ^18^, ^19^, ^20^ Although a majority of patients seems to correctly interpret PROMs information (displayed through various formats), a substantial portion (10%‐30% across studies) continues to experience trouble with interpretation, resulting in an inaccurate understanding of the information.^20^, ^21^, ^22^, ^23^

Health professionals are somewhat positive about using PROMs.^14^ However, they often do not refer to PROMs during their routine consultations with patients or do this only on an ad hoc basis.^24^, ^25^, ^26^ Barriers they experience include a lack of knowledge on how to use PROMs and lack of an adequate infrastructure for data collection and use.^6^, ^27^ The notion that PROMs such as a quality of life rating scales may be useful at an aggregated level as a benchmark to compare intuitions, but are perhaps less suitable (and were originally never designed to) guide decision making for individual patients, feed concerns or even resistance among health professionals towards using PROMs in consultations, thus creating important barriers for integration into daily clinical practice.^9^, ^28^

So, although there seems to be potential for using PROMs during routine medical encounters, there are also several issues that need further investigation. In particular, there is a lack of understanding what types of PROMs may be suitable for use during routine medical consultations and what type of presentation format would make information derived from PROMs understandable and meaningful for both patients and health professionals. Also, the contrasting perspectives of patients and professionals need further investigation.

Therefore, we here aimed to investigate: (a) how patients and professionals think about using PROMs during routine medical consultations (ie their perceptions, evaluations and comprehension); (b) for which purpose(s), patients and health professionals want to use PROMs during consultations; and (c) patients’ interpretation and use of PROMs in different presentation formats.

## 2 METHODS

This study used a cross‐sectional design, with Parkinson's disease as case example. For various reasons, Parkinson's disease can be regarded as exemplary for many other chronic progressive illnesses. First, Parkinson's disease is a chronic condition characterized by a wide range of both motor and non‐motor symptoms. Second, optimal management requires a multi‐disciplinary approach with involvement of a wide range of professional disciplines, involving all traditional echelons of care*.* The disease course is gradually progressive, with a lengthy time span of typically multiple decades. In the first part of our study, we conducted a series of semi‐structured interviews with people diagnosed with Parkinson's disease and with neurologists and physiotherapists providing care to people with Parkinson's disease. Based on these interviews, we identified key themes from the perspectives of both patients and professionals. This qualitative design allowed us to gain a better in‐depth understanding of the views of patients and to compare it in an efficient way to the professionals’ perspective. In the second part, we used these themes to build a survey. The survey was self‐administered by people with Parkinson's disease to assess their interpretation and perceived usefulness of different types of PROMs information. We chose to conduct this survey as a confirmatory study, allowing us to collect some quantitative evidence for the qualitative findings, especially concerning the interpretation of information in different formats. A patient representative from the Dutch Parkinson's Disease Association actively collaborated with us throughout the project. She commented on the interview guide, helped us recruiting patients for the interviews and was present during some of the interviews with patients. We also discussed the initial analyses of the interviews with the patient representative.

### 2.1 Semi‐structured interviews

#### 2.1.1 Participants

Patients (N = 13) were recruited in the Netherlands (Amsterdam area) through: (a) a local Parkinson's café (where patient representatives informed fellow patients about the study; N = 8); (b) an advertisement on the website of the Dutch Association of Parkinson's Disease (N = 1); and (c) the Neurology department of Amsterdam UMC, location VUmc (where one neurologist informed patients about the study; N = 4). No inclusion or exclusion criteria were used, except being diagnoses with Parkinson's Disease and being able to speak Dutch (inclusion). Interested participants could contact the research assistant (SV) and then received a patient information letter, which contained information about the goal and procedure of the study as well as about anonymity and confidentiality. Patients recruited by the neurologist were contacted by the research assistant. Next, an appointment was made to conduct the interview, either at a medical centre (N = 11) or at the participant's home (N = 2). Participants’ characteristics are shown in Table 1 (left column).

**Table 1:** Characteristics of people with Parkinson's disease participating in the interview study (N = 13) and survey study (N = 113)

Variable	Interview study N (%)	Survey study N (%)
Age		
<65 y	3 (23)	62 (55)
65‐75 y	6 (46)	33 (34)
≥75 y	4 (31)	2 (2)
Gender		
Men	4 (31)	61 (57)
Educational level		
Low (no or primary education)	3 (23)	14 (13)
Medium (secondary education)	5 (38)	34 (32)
High (tertiary education)	5 (38)	58 (54)
Disease duration		
0‐5 y after diagnosis	4 (31)	50 (47)
5‐10 y after diagnosis	5 (38)	33 (31)
>10 y after diagnosis	4 (31)	24 (22)
Health literacy (subjective)		
Adequate	4 (31)	NA
Numeracy (objective)		
Adequate	3 (23)	NA
Living situation		
At home—Single	NA	16 (15)
At home—With partner	NA	73 (68)
At home—With partner and children	NA	16 (15)
Hospitalized	NA	1
Country of birth		
The Netherlands	NA	104 (97)

NA, not available.

We recruited neurologists (N = 7; 2 women) and physiotherapists (N = 7, 4 women) within the Netherlands (Nijmegen area). Professionals were recruited through ParkinsonNet, a Dutch national network of professionals specialized in treatment and care of people with Parkinson's disease.^29^ Professionals within this network were invited to participate through email, followed by a reminder one week later to non‐responders. The researcher (MV) contacted professionals who responded. One physiotherapist was recruited through the network of one of the researchers in ParkinsonNet.

#### 2.1.2 Procedures

Interviews with patients were conducted by the research assistant (SV). The interviewer first again explained the study as well as anonymity and confidentiality, subsequently asked permission for audio‐recording, and then obtained informed consent. Next, the interviewer conducted the interview. The interviews had a total duration from about 45‐60 minutes. After the interviews, patients completed a short questionnaire about their socio‐demographic characteristics, health literacy^30^ and numeracy.^31^ Subjective health literacy was assessed based on the three subjective health literacy screening items developed by Chew et al^30^: (a) “How often do you have someone help you read hospital materials?"; (b) “How confident are you filling out medical forms by yourself?”; and (c) “How often do you have problems learning about your medical condition because of difficulty understanding written information?”. Inadequate health literacy if other answers than “never” on items 1 or 3 and/or other answers than “extremely” or “quite a bit” on item 2. Numeracy was assessed using one item from the Berlin Numeracy test developed by Cokely et al^31^: “Out of 1000 people in a small town, 500 are members of a choir. Out of these 500 members in the choir, 100 are men. Out of the 500 inhabitants that are not in the choir, 300 are men. What is the probability that a randomly drawn man is a member of the choir? Please indicate the probability in a percentage.” The correct answer is 25% and this was considered adequate numeracy; all other responses were considered to indicate inadequate numeracy.

Semi‐structured interviews with health professionals were conducted at different locations in several Dutch hospitals and physiotherapy practices. Five professionals were interviewed by telephone for logistic/pragmatic reasons. A researcher (MV) conducted the interviews with professionals. Professionals were provided with brief information about the study and were asked permission for audio‐recording. Next, the actual interviews were conducted (see measures). All interviews with professionals lasted between about 45 and 60 minutes.

#### 2.1.3 Interview topics

For all interviews, an interview protocol was used to assess participants’ perceptions of using PROMs in the medical encounter. The interview protocol for patients contained the following topics: current use of PROMs; comprehension of different types of PROMs information; explicit information needs; and preferred ways to receive PROMs information. Patients were also provided with different types of fictitious PROMs information, which were presented in different formats (ie line graphs, bar graphs and other visual formats). We distinguished between four main types of PROMs information: (a) individual PROMs scores; (b) individual PROMs scores with comparative data of similar patients; (c) aggregated PROMs scores for treatment options; and (d) aggregated PROMs scores for provider options. For each figure, the interviewer asked patients to explain the shown information in their own words and to evaluate their use of such information.

As for professionals, the interview protocol focused on the following topics: perception of pros and cons of discussing PROMs in routine medical consultations; types of PROMs that may be useful; preferences on how to communicate about PROMs; and factors that might influence their (non) use of PROMs data in medical consultations. To give professionals an idea of how PROMs information could look like, we provided several fictitious examples, based on the PDQ‐39, which is a disease‐specific quality of life instrument.^32^ Professionals were asked to reflect on these examples, for example to what extent they thought they would be useful and usable, and how they would explain the information to patients.

Both interview protocols were developed in an iterative way by the research team; OD and SV developed the draft version of the interview protocol for patients, and MV and MF developed the draft version of the interview protocol for professionals, addressing the main research questions. We did not use a specific conceptual or theoretical model, but rather used an explorative approach using broad concepts thought to be related to information needs and use (patients) and attitudes and perceived barriers/facilitators (professionals). These protocols were then exchanged between the researchers and refined further. Next, neurologists involved in Parkinson care and the patient representative involved in the study were asked to give feedback on the draft versions, both with respect to content and the way we formulated interview questions. After this feedback, the interview protocols were finalized.

#### 2.1.4 Data analyses

All interviews were transcribed literally. We employed qualitative thematic analyses with an inductive character. All transcripts were read and re‐read (14 interviews with professionals by MV and 13 interviews with patients by SV (patients)). Passages were selected, coded and related to our main topics in interview protocols. This resulted in two sets of codes (one for the professional interviews and one for the patient interviews), which were grouped into overarching themes. A subset of interviews (three patient interviews and six professional interviews) was coded by a second researcher (OD and MF) to ensure reliability of analysis. Disagreements between codes and/or identification of themes were resolved in separate consensus meetings between SV and OD and between MV and MF. We did not perform a member check nor a review by an independent analyst. However, we did involve the patient representative in the identification and interpretation of the themes. The analyses were conducted with the software program Atlas.ti.

The four people involved in the analyses were all researchers and not medical specialists, and had diverse backgrounds: one in decision psychology, two in health sciences and one in health psychology. They were all trained in qualitative research and in critically reflecting on their own role in interpretation of the data, and not again explicitly trained to do so in this specific study.

Quotes to illustrate the main themes were chosen after finalizing the analyses. Based on the main themes assessed, we searched for the underlying codes and corresponding quotes from the transcriptions. SV performed this selection of quotes for the patient interviews, under supervision of a senior researcher (OD), and MV performed this for the professional interviews, under supervision of a senior researcher (MF). Two researchers (OD and MF) made the final selection of the quotes for the manuscript.

### 2.2 Survey

#### 2.2.1 Participants and procedure

Participants were recruited through the user panel of ParkinsonNet (N = 221) and through announcements on two major Dutch websites on Parkinson's disease (ie ParkinsonConnect and the Dutch Association of Parkinson's Disease). The ParkinsonNet panel members received an invitation by email and a reminder after one week. Patients who were interested in participating after viewing the website announcements could directly access the survey through a web link. In total, 125 patients completed the survey. We excluded 12 cases because of poor data quality (ie no or hardly any questions were answered) resulting in a data set of 113 cases.

#### 2.2.2 Measures

**Figure 1:**
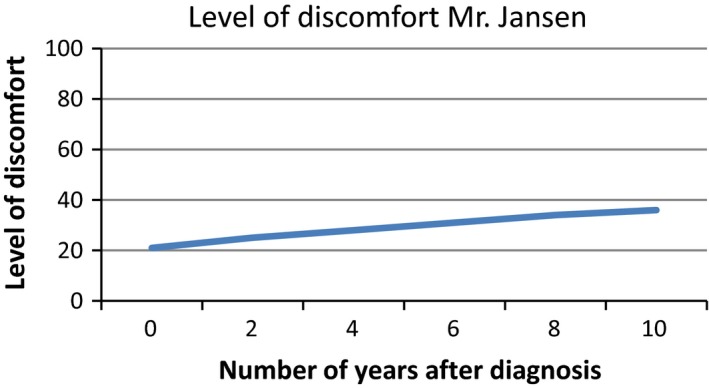
Individual PROMs scores over time, line graph

**Figure 2:**
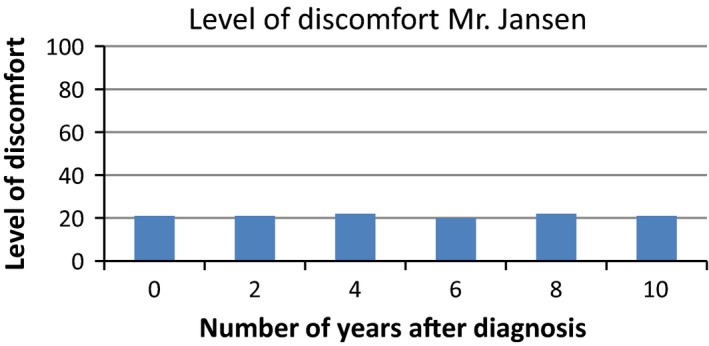
Individual PROMS scores over time, bar graph

**Figure 3:**
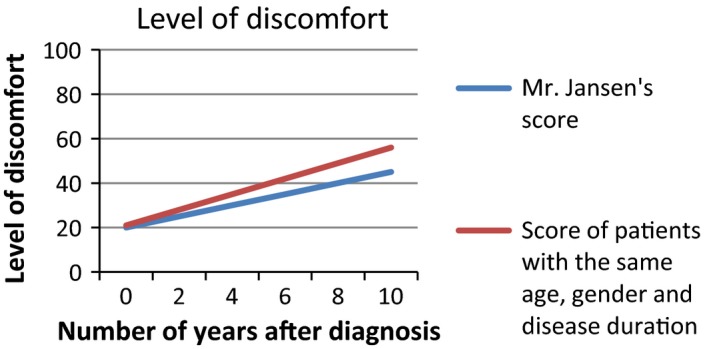
Individual PROMs scores with comparative data over time (ie average scores of similar patients), line graph

**Figure 4:**
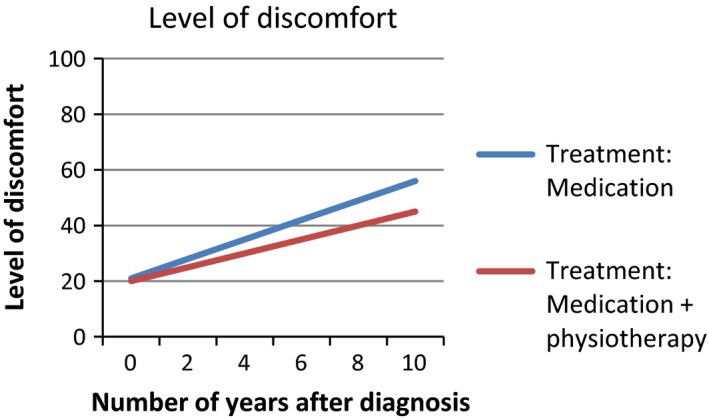
Aggregated PROMs scores over time with results of two treatment options, line graph

**Figure 5:**
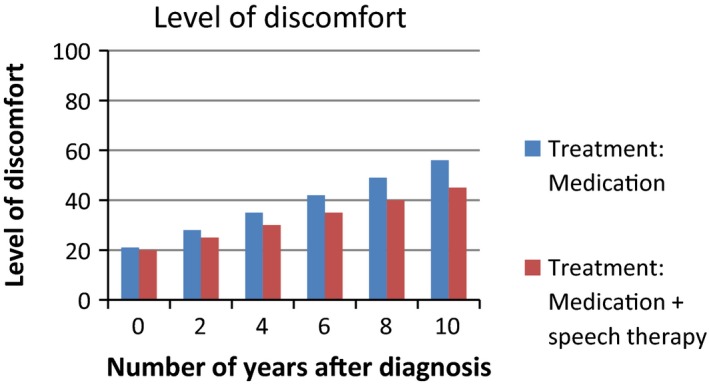
Aggregated PROMs scores over time with results of two treatment options, bar graph

**Figure 6:**
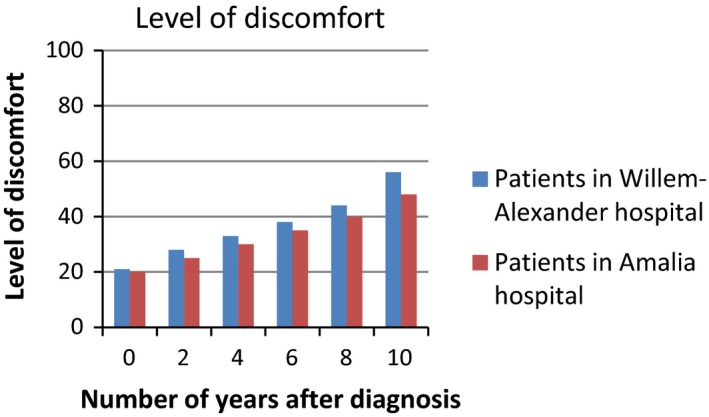
Aggregated PROMs scores over time with performance of two providers, bar graph

Based on the interviews with patients, we developed a survey to assess patients’ interpretation and use of PROMs in different presentation formats. We provided patients with six figures showing fictitious PROMs information (Figures 1, 2, 3, 4, 5, 6). We used the PROM “level of discomfort” as an example instead of “Quality of life” in the titles of the figures, to ensure alignment with the *higher = worse* directionality of the scores. All six figures were accompanied by explanations of the y‐as (0‐100 score, 0 = no discomfort and 100 = great discomfort) and x‐as (0‐10 years, 0 = time of diagnosis and 10 = 10 years after diagnosis).

After showing each figure, we posed several questions to assess the way patients interpreted and used the presented information. These questions were composed by the research team. First, we assessed *correct interpretation of the PROMs information* by using the following close‐ended question: “What does this figure say?” with multiple choice response options and one correct answer. Second, *perceived usefulness of the PROM*s information was assessed: “Would you find it useful to view this kind of information?” with response options (a) no, definitely not; (b) probably not; (c) maybe; (d) yes, probably; (e) yes, definitely. The exact wording varied for the six figures. Third, we assessed participants’ *attitude towards using the PROMs information in routine medical consultations*: “Would you like your health professional to discuss this kind of information during your medical routine consultation?” Response options were (a) yes and (b) no, and reasons for choosing yes or no were also assessed. We also assessed participants’ *preferences of presentation formats*; participants could choose between line graphs and bar graphs (ie Figure 1 vs Figure 2). For the figures that contained decision‐relevant information (Figures 4, 5, 6), we also assessed patients’ *hypothetical decisions*. Finally, socio‐demographic and disease‐related variables were assessed, such as age, gender, educational level, country of birth, living situation and disease duration.

## 3 RESULTS

### 3.1 Interviews with health professionals

We identified nine themes across the three interview topics (Table 2), with no distinct differences between neurologists and physiotherapists.

**Table 2:** Qualitative themes derived from the interviews with professionals (N = 14)

Interview topic	Themes and quotes
Perceived pros and cons of discussing PROMs, and types of PROMs considered useful	Theme 1: positive attitude towards individual PROMs scores over time “This is only signaling that it [change over time] is happening. And when you have noticed this, then you have to go deeper into it.” (physiotherapist, female)
	Theme 2: positive attitude towards using aggregated PROMs scores for treatment options “I think there are some patients who would like to have this information. Not everyone, but I think you can convince a number of people about this, if they have doubts or if they are looking for evidence." (neurologist, male)
	Theme 3: no positive attitude towards using aggregated PROMs scores for providers options “It is of course very interesting to benchmark yourself with other hospitals and then use that to see if you can improve yourself. In that sense I support it, but I don't find it useful for the patient.” (neurologist, male)
	Theme 4: perceived usefulness of individual PROMs scores with comparative data of similar patients “If they do better than average, then you can use it to empower the patient, they get positive energy from it. If they are doing worse, then it may be a signal, why are they doing worse? That is of course the intention, to put it that way. So that can certainly be useful.” (neurologist, male)
Preferred ways to communicate with patients about PROMs	Theme 5: questionnaires prior to encounter and actual information within‐encounter "[After filling out the questionnaires] Then you have a starting point, a basis for further discussion. And hence you have insight in the issues that arise, separate from physical decline.” (neurologist, female)
	Theme 6: patients should initiate discussions using PROMs “In the end, you just want patients to be able to access all their data [..]. They will eventually discuss it with their therapist. But first look at it on their own and think about it and then formulate questions and discuss them with a therapist.” (neurologist, male)
Factors influencing (non) use of PROMS data in routine medical consultations	Theme 7: availability of online tool “You should facilitate these kind of things digitally. What you need is a system where patients can, for example, enter online their scores. Then you do not have any extra work. Because the moment you have to complete it on paper and you have to make sure it is collected and you have to enter and process it [the PROMs information]... that will not work. So you would have to have it filled out on a website, for example, and then it should also be immediately available [during the encounter]." (neurologist, male)
	Theme 8: availability of training “I would like to have a lecture about it [the usage of PROMSs], so that it is not just my own interpretation, but also from others. It could be presented at a ParkinsonNet conference or something similar, with a speaker, that makes you think: ‘Now I get it’. … By applying it, you notice the added value of it….. You learn how to deal with it and learn how is can become part of the quality of care you provide.” (physiotherapist, female)
	Theme 9: positive attitude among professionals “For us, the PROMS are indispensable. Like I said, this is where we get most of our treatment goals from. Like I said, with this type of information, you trust your gut feeling. My colleagues do this too for themselves and so do I. So yes, that might differ from one another in how often and to what extent this will be provided.”(physiotherapist, male)

#### 3.1.1 Perceived pros and cons of discussing PROMs and types of PROMs considered useful

Health professionals had a strong preference for individual PROMs data over time. They thought this information could give patients insight into their disease progression, facilitate monitoring of treatment effects and facilitate personalized care and shared decision making (Theme 1). Professionals also identified the possibility to use aggregated PROMs scores as evidence for specific treatment options, in order to persuade patients to choose for particular options or to facilitate informed decisions of patients (Theme 2). In this context, PROMs information was thought to be a welcome supplement to clinical guidelines.

Although professionals generally agreed that aggregated PROMs scores for provider options could be useful for internal quality improvement, only few said they would use this information in consultations with their patients (Theme 3). This was largely due to a lack of trust in data quality as well as in patients’ capacity to judge the data at its true value, but also to their perception that patients do not want to use this information. Some professionals also perceived individual PROMs scores with comparative data of similar patients useful (Theme 4). According to these professionals, this information could contribute to improve disease knowledge and acceptance, and possibly also to offer hope and to encourage patients. However, some professionals expressed doubts regarding the usefulness of this comparative information. They thought that in case of lower than average scores, this information might be threatening for patients and would actually discourage them.

#### 3.1.2 Preferred ways to communicate with patients about PROMs data

Professionals clearly preferred patients to complete PROMs prior to a consultation. Professionals also preferred to receive and view PROMs on a computer screen to easily show the information to patients on the screen during the consultation (Theme 5). Professionals were reluctant to address PROMs information unsolicited during consultations and expected patients to take the initiative in discussing PROMs information (Theme 6). This was mainly because they thought patients would differ in their needs, depending on age, educational level, disease stage and personality, and that many would not want to receive or discuss PROMs information. When asked about presentation formats, professionals preferred line and bar graphs, and scores from repeated measurements over time, rather than data based on only one or two measurement moments. Professionals wanted to include multiple individual quality of life domains (eg mobility, emotional well‐being) in their discussions, rather than one overall quality of life score.

#### 3.1.3 Factors influencing (non) use of PROMS data in the medical encounter

Professionals perceived the availability of an online portal, in which patients could fill out PROMs and receive the results, as the most important factor enabling the use of PROMs data during routine consultation (Theme 7). In addition to such a “within‐encounter” tool, professionals also saw possibilities for patients to review PROMs data in a “pre‐encounter” tool. Professionals felt the need for training in PROMs in general and more specifically in how to use PROMs during consultations with patients (Theme 8). Finally, appearing more indirectly from the data was the need to have a positive attitude towards using PROMs in consultations (Theme 9). Several professionals appeared to be sceptical towards the quality and reliability of PROMs as health‐care quality measurements. In this respect, several professionals also emphasized the need for role models and pioneers working in the field of Parkinson's disease.

### 3.2 Interview study patients

We identified 12 themes across the four interview topics. These are listed in Table 3, illustrated with patients’ quotes.

**Table 3:** Qualitative themes and subthemes derived from the interviews with patients (N = 13)

Interview topic	Themes and quotes
Current use of PROMs	Theme 1: Medical encounter is concentrated on medication regimen, and not on treatment decisions in general “Well, how far should such a neurologist go? It's only 15 min. (..) The neurologist is more like a medication person.” (female, 43 y, highly educate, disease duration of 6 y) “I walk and do exercises with that hand, and then she comes in and advises a pill or another pill.” (Male, 70 y, highly educated, disease duration of 6 y)
	Theme 2: Patients do fill out PROMs questionnaires, but they do not remember the data being discussed by professionals Patient: “Indeed, I filled out such a questionnaire. (..) On my PC at home. Yes, He < neurologist>sent it to me." Interviewer: "And did your doctor discuss the information afterwards with you?” Patient: "No.” (Female, 52 y, medium educated, disease duration of 10 y) “No I don't believe that we discussed it.” (Female, 77 y, highly educated, disease duration of 3 y) “No, not with the neurologist (..). I did so with my oncologist but that was 7 y ago.” (Female, 66 y, medium educated, disease duration of 3 y)
	Theme 3: Mixed preferences as to discussing PROMs data with professionals “Because it offers an entrance to talk. I really think that I would start on that myself, at that moment.” (Female, 52 y, medium educated, disease duration of 10 y) “About quality and life and yes.. Well, … we're far from that. That's why I say: I am busy with the things I'm still able to do.” (Male, 71 y old, medium educated, disease duration of 3 y)
Comprehension of PROMs data	Theme 4: Gist of PROMs data is adequately comprehended, but not the exact details <this means> “that hospital two has a better score than hospital one.” <patient responding to comparative hospital information, only focusing on comparison but not on dimension on which hospitals are compared> (Male, 71 y old, medium educated, disease duration of 3 y) <this means> “well, that I was quite ill.. that's what I think. Or is that incorrect?”<patient responding to individual PROMs scores on ‘pain’, focusing on the general concept of ‘illness’ and not on the Quality of Life dimension ‘pain’> (Female, 66 y, medium educated, disease duration of 3 y) “I would choose the red line.” Interviewer: "the combination of medication and occupational therapy?" Patient: "Yes" <patient responding to choice‐information about treatment options, only focusing on one option and on general concept of Quality of Life (Female, 72 y, highly educated, disease duration of 6 y).
	Theme 5: Use of higher = worse directionality hindered comprehension “Well, I really have to study on the fact that it goes the other way around.”(Female, 75 y, medium educated, disease duration of 3 y) “This increases.. Oh no, that's not the way I should describe it.” Interviewer: "No, a lower score is better actually". (Female, 66 y, medium educated, disease duration of 3 y)
	Theme 6: Comparative anchors to indicate the scores of others difficult to interpret and use “Because it indicates what I'm busy with. So that I'm on the right track. Oh wait.. I'm not on the right track here, right?” <patient misinterpreting the information using a comparative anchor> (Male, 71 y old, medium educated, disease duration of 3 y)
	Theme 7: Line and bar graphs outperformed other formats, both for comprehension and patient preference “When you have both medication and occupational therapy, mobility will worsen less compared to using only medication.” <patient correctly responding to information in a line diagram> (Female, 52 y, medium educated, disease duration of 10 y)
Explicit information needs	Theme 8: Clear interest in PROMs in context of treatment decisions, compared to PROMs in context of provider choice, nor for individual PROMs scores over time “But it may well be that I need adjustments in the future. Based on this figure, I would not hesitate to opt for ergo therapy.”(Female, 52 y, medium educated, disease duration of 10 y) “I like to compare things with each other.” (Male, 76 y old, medium educated,unknown disease duration). “I would not want such information. That would not help me. (..) I know myself how I feel and what I am doing.” <patient responding to individual scores (Female, 66 y, highly educated, disease duration of 3 y) “Why would it help me to know that my situation has greatly worsened?” <patient responding to individual scores over time> (Female, 68 y, low educated, disease duration of 11 y)
	Theme 9: All quality of life dimensions important “They are all important. Imagine that I would become a bit of a zombie or that I would get depressed. Or that I could not leave my house, or could not easily move through my house? Yes, I would find all those aspects important.”(Female, 78 y, highly educated, disease duration of 8 y) “They are all.. all six <dimensions>, they are all related to each other. And this one.. I also found this one important.” (Male, 72 y, highly educated, disease duration of 6 y) “Informal care is also very important of course. Emotions as well. Communication.. I cannot choose, I find them all important..” (Female, 77 y, highly educated, disease duration of 3 y)
Preferred ways to receive PROMs data	Theme 10: Clear need to discuss PROMs data directly after diagnosis “When someone receives a diagnosis, it is very important to get all the information immediately.” (Female, 43 y, medium educated, disease duration of 2 y)
	Theme 11: Professional should initiate discussions about PROMs in the medical encounter “It would be very good if the doctor would immediately enter into the conversation. If I would receive the information in another way, then I would be the imitator again, to open the conversation about it. That is not my strongest suit.”(Female, 52 y, medium educated, disease duration of 10 y) “The doctor, so you can immediately discuss it together.” (Male, 70 y, low educated, disease duration of 16 y)
	Theme 12: Preference to discuss PROMs data with neurologist or nurse, and then to reconsider it at home “That the neurologist would discuss it with me. And then I would like to bring it with me at home.” (Female, 78 y, highly educated, disease duration of 8 y) “I would want to hear this from a medical doctor.” (Male, 76 y, medium educated, unknown disease duration) “To hear it from your neurologist. (...Yes, I have a good relationship with my neurologist. So in that sense, I would like to discuss it with him.” (Female, 72 y, highly educated, disease duration of 6 y).

#### 3.2.1 Current use of PROMs

Patients reported there was little time for discussions about treatment options during routine medical consultations, and they experienced professionals (especially neurologists) to focus only on the medication regimen in the time available (Theme 1). Most, but not all patients remembered having completed quality of life questionnaires; however, the derived data were not discussed with them during consultations (Theme 2). Patients expressed mixed preferences regarding discussing PROMs data with professionals (Theme 3). Some patients mentioned that PROMs could help start a conversation with their health professionals about topics prioritized by themselves. Others stressed that they were not interested in discussing PROMs with their health professional, mainly because the topic quality of life was not of interest to them right now.

#### 3.2.2 Comprehension of different types of PROMs data

Overall, patients adequately comprehended the PROMs information we showed them in terms of their gist meaning, but not in terms of the exact details (Theme 4). For example, most patients understood that a certain treatment (eg medication with physiotherapy) was better than another treatment (only medication), without including what Quality of life dimension was actually the object of comparison in their consideration. Two specific aspects particularly hindered easy comprehension: (a) the use of a “higher = worse” directionality (Theme 5) and (b) the use of comparative information of patients that are similar in terms of age, gender and disease progression (Theme 6). Although participants in general understood the directionality of the information after clarification provided by the interviewer, it nevertheless hindered them in reading and deriving meaning from the information. The fact that *higher* Quality of life scores indicated *worse* Quality of life was counterintuitive for most patients and caused difficulties in interpreting the scores. Similarly, comparative data were difficult to interpret and caused confusing for some patients because they had to keep in mind two scores and perceived the comparative data as their own or vice versa. Patients interpreted PROMs information most often correctly when line and bar graphs were used, compared with more evaluative formats such as smileys and colours (Theme 7).

#### 3.2.3 Explicit information needs

After being probed, participants showed a clear interest in PROMs information for treatment decisions, compared with PROMs information for provider choice or monitoring of individual disease progression over time (Theme 8). This information, but also the more individual scores, seemed to motivate participants to think of behavioural options, such as what they could do themselves to improve their Quality of life. All Quality of life domains presented were considered important by participants (Theme 9). From all PDQ‐39 domains (mobility, emotional well‐being, cognition, communication, physical discomfort, load for informal caregivers), participants found mobility and physical discomfort the most informative and important domains.

#### 3.2.4 Preferred ways to receive PROMs data

There was a strong preference to receive the information as soon as possible (ie immediately after diagnosis; Theme 10) and from their health professional (Theme 11). Neurologists and nurses were the preferred health professionals to receive information from (Theme 12). According to most patients, their health professional should initiate a conversation about PROMs. Some patients explicitly stated that they did not feel equipped to start this conversation themselves. Most patients also preferred the possibility of taking the PROMs information home so they could reconsider the information after having discussed it with their neurologist or nurse.

### 3.3 Survey patients

Table 1 (right column) describes characteristics of people with Parkinson's disease who participated in the survey (N = 113). A substantial number of participants did not complete the full survey and dropout seemed to be a function of the survey length; response rates for the initial questions of the survey were relatively high (only 7.1% missing for the first presentation format), whereas response rates increased towards the end of the survey (25.7% missing for sixth presentation format). Table 4 displays descriptive findings relating to our main variables, only for those participants who filled out the corresponding questions.

**Table 4:** Comprehension and perceived usefulness of, attitude towards and hypothetical decisions based on PROMs information depicted in six different formats (Figures 1, 2, 3, 4, 5, 6) among people with Parkinson's disease (N = 113)

Variable	Figure 1: Individual PROMs scores over time, line graph	Figure 2: Individual PROMs scores over time, bar graph	Figure 3: Individual PROMs scores with comparative data over time (ie average scores of similar patients), line graph	Figure 4: Aggregated PROMs scores over time with results of two treatment options, line graph	Figure 5: Aggregated PROMs scores over time with results of two treatment options, bar graph	Figure 6: Aggregated PROMs scores over time with performance of two providers, bar graph
Comprehension (% correct answer)	78 (74.3%)	86 (87.8%)	72 (74.2%)	82 (90.1%)	77 (87.5%)	68 (81.0%)
Perceived usefulness of information (% who finds the information certainly useful (score of 5 on scale from 1 to 5)	46 (43.8%)	43 (43.9%)	Not asked for this figure	51 (56.0%)	42 (47.2%)	36 (42.9%)
Attitude as to use in medical encounter (% who does want to use PROMs information)	95 (90.5%)	88 (89.8%)	82 (84.5%)	86 (94.5%)	82 (92.1%)	69 (82.1%)
Hypothetical decision (% correct answer)	‐	‐	‐	83 (91.2%) choose medication + physiotherapy	70 (78.7%) choose medication + speech therapy	54 (64.3%) choose Willem Alexander hospital

#### 3.3.1 Correct interpretation of PROMs information

Overall, most patients interpreted the PROMs information reasonable to very good. Correct interpretation across the provided figures varied from 74.2% (Figure 3, showing individual PROMs scores with comparative data of similar patients over time, depicted as a line graph) to 90.1% (Figure 4, showing aggregated PROMs scores over time with results of two treatment options, depicted as a bar chart). Individual PROMs scores over time were interpreted more often correctly when presented in a bar graph (Figure 2; 87.8% correct) compared to a line graph (Figure 1; 74.3% correct). Bar graphs were also preferred by patients (57.2%) compared to a line graph (42.3%).

#### 3.3.2 Perceived usefulness

Patients perceived the figure showing aggregated PROMs scores over time with results of two treatment options presented in a line graph as most useful (Figure 4; 56% of patients found this information certainly useful), followed by the figure showing this same information in a bar graph (Figure 5; 47% found this information certainly useful). Aggregated PROMs information with performance of two providers was perceived as least useful (only 43% found this information certainly useful; Figure 6).

#### 3.3.3 Attitude towards use of PROMs information in routine clinical consultations

Patients were most positive towards using the aggregated PROMs scores comparing two treatment options in medical encounters with their professionals (94.5% of participants said they wanted to discuss Figure 4 and 92.1% to discuss Figure 5). The main reasons why patients wanted to discuss these scores were that it gave them insight into the effects of treatment (25.6% and 32.9% for Figures 4 and 5, respectively) that it facilitated them in making treatment decisions (27.9% and 24.4% for Figures 4 and 5, respectively) and in participating in decisions with health professionals (26.7% and 28.0% for Figures 4 and 5, respectively).

#### 3.3.4 Hypothetical decisions

We asked participants which treatment options they would choose (eg medication with physiotherapy or medication only) based on figures showing PROMs information relevant for decision making (Figures 4, 5, 6). Between 78.8% and 91.2% of the participants chose the option with the best treatment effects as shown in the figures. Especially, the line graph showing the results of two treatment options (Figure 4) resulted in decisions reflecting adequate comprehension of information. The vast majority (91.2%) said to choose medication and physiotherapy, which indeed showed the best treatment effects in Figure 4. For similar scores presented in a bar graph (Figure 5), only 78.7% chose the correct combination of medication and, in this case, speech therapy.

## 4 DISCUSSION

We investigated the views of people with Parkinson's disease and their health professionals about using PROMs during routine medical consultations. Although both patients and health professionals were overall positive about using PROMs, we demonstrated conflicting expectations regarding who should initiate a conversation about PROMs during the consultation. Importantly, patients and professionals had a different preference for the type of PROMs information. Specifically, individual PROMs scores over time were mostly preferred by professionals, whereas aggregated PROMs scores for different treatment options were mostly preferred by patients. Interpretation of PROMs information by patients was reasonable to good, depending on the presentation format. Bar graphs were most often interpreted correctly and were also the preferred presentation format for patients.

### 4.1 Strength and limitations

A limitation is that we used convenience samples of patients and professionals, which may limit the generalizability of the findings. Qualitative interviews were performed with 13 patients, and the question remains whether their perspective also reflects the more general perspective of Dutch people with Parkinson's disease. We were however able to include a diverse group of patients, both with respect to disease duration and educational level, which decreases the chance of systematic selection bias. Among the health professionals, there were no distinct differences in the views of neurologists vs physiotherapists, which also supports the notion that that there was no great selection bias. Of concern is that a quarter of our participants did not complete the full survey, which is also a more general concern when using web‐based questionnaires^33^ and which may impact the external validity of our findings. Both for the interviews and the survey, it might be that patients had trouble placing themselves in our hypothetical scenarios and information. Furthermore, although the study aims were generic in nature, we only included people with Parkinson's disease and professionals working in Parkinson care (noting that Parkinson's disease is good model condition, as outlined in the introduction). It is certainly relevant to repeat the study in settings for other chronic conditions and using patients’ actual PROMs scores. Nevertheless, a major strength of this study was the mixed‐methods design and the integration of multiple perspectives (both patients and health professionals) in the analysis. Furthermore, unlike a previous study about the use of PDQ‐39 in routine care,^34^ we used various presentation formats covering all purposes for which PROMs information can be used.

### 4.2 Discussion of findings

Both qualitative and quantitative findings showed that people with Parkinson's disease mainly embraced the use of aggregated PROMs for decisions about treatment options. For this type of information, we found the highest perceived usefulness as well as the most positive attitude among patients. Qualitative data suggested that patients are positive about this particular type of information because it motivates them to think about different possibilities to improve their Quality of life, such as physiotherapy, occupational therapy and medication options. Corroborating the objectives of shared decision making, it thus seems that providing patients with PROMs information (just like providing them with clinical outcomes data) can have a role in becoming more knowledgeable and more active in decision making.

Whether shared decision making based on PROMs will really occur will, however, likely depend on the role that professionals take on in consultations. Our interviewed patients relied on the initiative of professionals to actually discuss PROMs data, which aligns with previous study findings in various clinical settings.^35^, ^36^, ^37^, ^38^ Patients are interested in information about treatment options and in shared decision making,^39^, ^40^ but also tend to leave the initiative in the consultation largely to health professionals when they are not explicitly invited to collaborate.^41^, ^42^ Because professionals in this study indicated that patients should take the initiative in discussing PROMs, it seems somewhat unlikely that PROMs information will actually be used for shared decision making, unless professionals will be explicitly instructed and trained in taking the initiative. In addition, initiatives to stimulate an active role of patients may be needed.^13^

Correct interpretation of the PROMs information in our survey study varied from 74% to 90% across presentation formats. This finding is similar to previous findings in this field. For example, McNair et al^22^ found accuracy rates ranging from 85% to 98% across six formats in a sample of 192 patients. Others found similar percentages.^23^, ^43^, ^44^ Interpretation in our study seemed to be most accurate when using a bar chart compared to a line graph, although it should be said that one particular figure using a line graph (ie aggregated PROMs scores over time with results of two treatment options) was also accurately interpreted. Therefore, it seems to depend partly on the type of information presented, which format was associated with better comprehension. The finding that especially bar charts support user understanding seems to be somewhat in contrast with the findings of previous studies showing that line graphs are especially suitable for PROMs presentation.^21^, ^43^ However, as recently stated by Tolbert and colleagues,^20^ no single format seems to be best for all patients in all situations. Studies comparing presentation formats of quantitative information for treatment decisions in general have often depicted bar charts as a suitable presentation format.^45^, ^46^, ^47^, ^48^ Bar charts are known to especially support the ease of making relatively simple comparisons between groups,^47^, ^49^ which may well explain why they resulted in correct interpretation in our study. Tailored presentations of PROMs information, based on patients’ own preferences, may become facilitated by the use of apps to collect and display PROMs, for example in value‐based health‐care initiatives.

## 5 CONCLUSION

Overall, the findings of our study confirm that both patients and professionals are positive towards the use of PROMs information in routine medical consultations. However, we found conflicting expectations and preferences of patients and professional as to who should initiate the conversation about PROMs information and which types of PROMs should be prioritized in those conversations. Overall, patients interpreted the PROMs information reasonably well, especially when presented through bar or line graphs.

## ETHICAL CONSIDERATIONS

The study protocol was approved by the Medical Ethics Review Committee of Amsterdam UMC, location VUmc (file number 2016.049).
